# Antisense oligonucleotide-mediated exon 27 skipping restores dysferlin function in dysferlinopathy patient-derived muscle cells

**DOI:** 10.1016/j.omtn.2025.102462

**Published:** 2025-02-04

**Authors:** Naoki Suzuki

**Affiliations:** 1 Department of Rehabilitation Medicine, Tohoku University Graduate School of Medicine, Sendai, Japan; 2 Department of Rehabilitation, Tohoku Kosai Hospital, Sendai, Japan

## Main text

Dysferlinopathy is a progressive muscular disorder caused by the deficiency of dysferlin, a protein crucial for muscle membrane repair. Until now, there has been no effective treatment, significantly impacting patients’ quality of life. In a recent issue of *Molecular Therapy Nucleic Acids*, Anwar et al. successfully restored dysferlin function using an exon 27 skipping therapy with antisense oligonucleotides (ASOs).^1^ This study demonstrated functional recovery in muscle cells derived from dysferlinopathy patients. This achievement marks a significant step toward developing a treatment for dysferlinopathy and suggests potential applications for other hereditary muscle diseases. However, further investigation is needed for clinical application, including *in vivo* efficacy verification and long-term safety assessment.

Dysferlinopathy is a type of muscular dystrophy with autosomal recessive inheritance, typically onset in a person’s late teens to 20s. The dysferlin protein plays an essential role in muscle cell membrane repair, and its deficiency leads to muscle fiber degeneration and necrosis. The dysferlin gene is located on chromosome 2p13.3-p13.1 and is composed of 55 exons coding for 2,080 amino acid residues, which form the dysferlin protein.^2^ Dysferlin is involved in calcium-mediated membrane fusion events and plasma membrane repair.^3^ Currently, there is no fundamental treatment for dysferlinopathy, with supportive therapies such as symptomatic treatment and physical therapy being the main options.

In recent years, ASO therapy has gained attention as a new approach for genetic diseases. ASOs are short nucleic acid molecules that bind to specific mRNAs to control splicing or regulate protein expression. Some ASO drugs, such as Spinraza (nusinersen) and Viltolarsen (eteplirsen), have already been approved, demonstrating their efficacy and safety.

Anwar et al. designed an ASO to skip exon 27 of the dysferlin gene. Exon 27 is known to be less essential for dysferlin protein function, and skipping this exon can avoid frameshift mutations and produce partially functional dysferlin. The research team verified the effect of ASO treatment using muscle cells derived from dysferlinopathy patients. As a result, exon 27 skipping restored dysferlin protein expression and improved muscle cell membrane repair ability. The authors also demonstrated that the ASO therapy reduces cytotoxicity and apoptosis in patient-derived muscle cells, along with positive changes in cell vitality.

In their prior work, Anwar et al. demonstrated that dysferlin-deficient patient-derived fibroblasts partially retained membrane resealing functionality when transfected with dysferlin constructs lacking exons 26–27 or 28–29, producing an internally truncated dysferlin protein.^4^ This suggests that certain dysferlin exons may be dispensable for essential membrane repair function. Exon pairs 26–27 and 28–29 specifically code for parts of the DysF domain, a region implicated in muscular dystrophies due to its frequent mutation,^5^ although its precise functional role is still under investigation. Targeting these regions for exon-skipping therapies could address a broad spectrum of mutations within this domain.

However, several important challenges remain for clinical application. *In vivo* efficacy verification is essential. Experiments using animal models, especially dysferlin-deficient mice, are necessary to confirm the effects and safety of ASO therapy *in vivo*. For instance, Malcher et al. demonstrated pathological and functional improvements *in vivo* using U7-based exon skipping in an exon 38 missense mouse model (p.Leu1360Pro).^6^ The lack of an appropriate mouse model with apparent motor function decline is also a challenge hindering *in vivo* verification of exon skipping.

Additionally, it has been pointed out that various proteins bind to the DysF domain. Ono et al. reported that AMP-activated protein kinase (AMPK) binds to the region containing the DysF domain, that AMPK plays an important role in membrane repair function, and that AMPK activators improve dysferlinopathy models.^7^ Recent analyses using cryoelectron microscopy have indicated that the DysF domain plays a crucial role in dimer formation and protein stability.^8^ The impact of exon skipping on the function of dysferlin-binding proteins and long-term safety remains an issue to be verified *in vivo*.

Despite these remaining challenges, the research of Anwar et al. has opened up new possibilities for dysferlinopathy treatment. The restoration of dysferlin function through exon 27 skipping could serve as an important bridge from basic research to clinical application. It is expected that after *in vivo* verification, clinical trials will proceed, expanding treatment options for dysferlinopathy patients.

Furthermore, this research approach may influence the development of treatments for other hereditary muscle diseases. With the advancement of ASO therapy, it is anticipated that innovative treatments for more intractable diseases will be developed. The progress in dysferlinopathy research reaffirms the importance of developing treatments for rare diseases and heralds the dawn of a new era in gene therapy.

## Declaration of interests

The author declares no competing interests.
